# Health-Related Quality of Life Measures for Physically Active Elderly in Community Exercise Programs in Catalonia: Comparative Analysis with Sedentary People

**DOI:** 10.1155/2013/168482

**Published:** 2013-12-24

**Authors:** Jesús Fortuño-Godes, Myriam Guerra-Balic, Josep Cabedo-Sanromà

**Affiliations:** ^1^Esport 3, 08001 Barcelona, Spain; ^2^Facultad de Psicología, Ciencias de la Educación y del Deporte Blanquerna, Universidad Ramón Llull, Departamento de Ciencias de la Actividad Física y Deporte, 08022 Barcelona, Spain

## Abstract

*Objective*. To evaluate Health-Related Quality of Life (HRQoL), medication used, and Stock of Health Capital (SHC) in physically active elderly participants in Community Exercise Programs (CEPs) compared to a sedentary group. *Methods*. EuroQol standardized instrument was completed by physically active elderly (*n* = 2,185) who participated in CEPs. Common items were compared to HRQoL data of 1,874 sedentary elderly people, taken from the Catalan Health Survey 2006 (CHS'06). Visual Analogue Scale (VAS) outcomes and medication used were assessed through parametric statistics. Dimensions of health conditions were compared, between sedentary people and physically active elderly participants in CEPs. SHC results were obtained combining the EuroQol scores and Life Expectancy (LE) values. An economic value of €34,858.70 was assigned to these years of LE. *Results*. Physically active subjects had better HRQoL values (75.36 in males and 70.71 in females) than CHS'06 sedentary subjects (58.35 in males and 50.59 in females). Medication used was different between physically active subjects (1.89 in males and 2.87 in females) and CHS'06 sedentary subjects (4.34 in males and 4.21 in females). SHC data for physically active elderly (€465,988.31/QALY in males and €522,550.31/QALY in females) were higher than for CHS'06 sedentary subjects (€363,689.33/QALY in males and €346,615.91/QALY in females).

## 1. Introduction

Regular physical activity increases functionality of the body's biological dimensions and is associated with lower mortality rates (US Department of Health and Human Services, 2000) [1]. There is clear evidence that physical activity delays mortality in comparison to inactivity [2].

Research evidence also shows an association between physical activity and psychological well-being, such as a reduction in depression and anxiety or increase in levels of self-esteem and positive affect [3].

### 1.1. Concept of HRQoL and Its Application in the Field of Physical Activity for the Elderly

An individual's health has been traditionally measured on the basis of indicators of morbidity, mortality, and Life Expectancy. At present, there is a search for new, more dynamic ways to measure health, with an emphasis on the subjective dimension. The World Health Organization, in its institutional definition, already established this tendency when defining health as a state of absolute physical, mental, and social well-being, rather than just the absence of disease [4]. The concept of Quality of Life (QoL), in this respect, becomes an element to be considered in research on particularly vulnerable groups such as the elderly. And the individual's perception of their own health condition, understood according to the concept of Health-Related Quality of Life (HRQoL), has become significantly more and more relevant [5, 6]. The changes observed often do not relate to indicators, particularly to physical fitness [7]. On many occasions, there is no clear relation between HRQoL changes and fitness [8].

Gusi et al. [9] applied HRQoL measures using the EuroQol scale on subjects participating in a six-month walking program. Results were used to assess cost utility (CU) of benefits observed. The mean treatment cost per patient in the group was €41 more than “best traditional care” in primary health care at the end of the program.

Furthermore, health can also be understood as an investment, when applying prevention measures. Health is, from this perspective, a kind of Human Capital [10]. A person's Stock of Health Capital (SHC) represents their health's updated value for the rest of their life [11]. Every person has an initial Health Capital, which is devalued with age and may be increased through those resources arranged by public institutions (health, social, and sports resources) or by engaging in behaviors related to healthy habits such as diet, physical exercise, or natural environment.

In Spain, SHC studies have been applied to population in Catalonia [12] by using data from the Catalan Health Surveys (CHS) corresponding to years 1994 and 2006 [11] and in the Canary Islands during 2004 [13]. These studies applied the concept of SHC in large samples of population without considering the level of exercise that their subjects engaged in.

So, the aim of the present study was to assess SHC, HRQoL, and medication used in a sample of physically active elderly subjects engaging in a community-based physical exercise program in comparison to a group of sedentary elderly in Catalonia, derived from the 2006 Catalan Health Survey.

## 2. Methods

The sample consisted of a group of individuals, older than 60, who participated in exercise programs, including activities such as keep-fit, tai chi, walking, pétanque, and water activities. In total, 2,185 subjects took part in the study (356 males and 1,829 females) from October 2004 to May 2007. Sampling was intentional, because it was known by the main researcher. Participants came from the four provinces of Catalonia (Tarragona, Girona, Barcelona, and Lleida). All the groups participated in Community Exercise Programs (CEPs) that had a common protocol of activities, intensity, and frequency.

Data for the sedentary population was taken from the Catalan Health Survey (CHS) conducted in the year of 2006. This survey was organized by the Government of Catalonia (Generalitat). Data from CHS'06 were transferred by the Health Department of Generalitat. In total, there were 1,874 subjects (759 males and 1,115 females). Data were classified according to health geographic region. Health care resources did not change along the period of study. A database was generated with the results from the elderly participating in the CEPs and from the sedentary subjects, with a total number of 4,061 participants. Age and gender differences were analyzed separately.

Both surveys, the present one and the CHS one, were anonymous and voluntary.

### 2.1. Measurement Instruments

Measurement instruments were divided into three separate parts: sociodemographic data, objective health measures (number of illnesses and medication used), and HRQoL measure. Questionnaires were distributed and filled in individually by physically active elderly during group sessions with the supervision of a research assistant.

To assess HRQoL, the EQ-5D questionnaire was used [14, 15], as was the case for the CHS'06. This questionnaire has three different parts:

1. descriptive system (ds), with five dimensions: mobility, self-care, usual activities, pain/discomfort, and anxiety/depression.
2. Visual Analogue Scale (VAS), with scores from 0 to 100, with end points labeled as “*worst imaginable health state*” and “*best imaginable health state*”;
3. health costs. EuroQol has a system to transform DS into costs or preferences of population for all the health states.

To calculate SHC, results from the EuroQol costs were used, multiplying results of preferences by Life Expectancy (LE). This value is labeled as QALY (Quality Adjusted Life Year) [11]. Subsequently, an economic value of  €34,858.70 per year was assigned to these years of life, following a method described by Sacristán et al. [16] and used in other studies to calculate cost utility in sports practice [9].

### 2.2. Data Processing

Descriptive statistics were obtained for all the variables. Values corresponding to mean VAS of EuroQol, medication used, and SHC, in relation to gender and age, were analyzed using Student's *t*-test. A statistical homogeneity test was not positive. An alpha level of 0.05 was selected to consider that the difference of means was significant. Data analyses were carried out using the statistical package SPSS, version 15.0 for Windows.

## 3. Results

### 3.1. HRQoL Assessment

VAS results showed that the men and women of the sample corresponding to the group of physically active elderly have a significantly (*P* < 0.05) higher HRQoL (75.36 and 70.71) than CHS'06 sedentary men and women (58.35 and 50.59), in all the age groups analyzed (Table 1).

Men have higher HRQoL than women, both in physically active and sedentary subjects.

The HRQoL Assessment decreases with age in physically active elderly females (from 73.30 in the 60-to-64 group to 62.11 in the older-than-85 group), sedentary males (from 62.5 in 60-to-64-year-old to 52.56 in older-than-85 subjects), and sedentary females (from 56.17 in 60-to-64 group to 49.25 in older-than-85 group). This tendency is similar in physically active elderly males from 60 to 64 (76.81) to 75 to 84 (74.91), as shown in Table 1.

### 3.2. Assessment of Medication Used

In the case of elderly men, physically active subjects have a significant lower mean for medication used with 1.89 units in comparison with sedentary men (4.34 units), in all the age groups analyzed. Physically active females showed a significant difference too (2.87) with sedentary women (4.21) in all the age groups (Table 1).

Women use more medication than men when being physically active and sedentary.

Medication used increases with age in physically active elderly males (from 1.21 in the 60–64 group to 2.19 in older than 85), physically active elderly females (from 2.67 in 60–64 to 2.77 in 75–84 group), sedentary males (from 4.11 in 60–64 to 4.67 in 75–84), and sedentary females (from 4.21 in 60–64 to 4.52 in 75–84), as shown in Table 1.

### 3.3. Stock of Health Capital

The SHC of physically active elderly (€465,988.31 for males and €522,550.31 for females) is higher than the one of the sedentary subjects (€363,689.32 for males and €346,615.91 for females) in all the age groups (Table 1).

Men have higher SHC than women when being sedentary, but this tendency is the opposite when being physically active. Physically active females showed higher SHC than physically active males.

SHC decreases with age in physically active males (from €671,536.21 in the 60–64 group to €172,838.28 in older than 85), physically active females (from €740,420.35 in 60–64 to €328,150.81 in 75–84), sedentary males (from €600,250.46 in 60–64 to €107,375.55 in older than 85), and sedentary females (from €319,454.92 to €167,701.16 in the 75–84 group), as shown in Table 1.

### 3.4. Assessment of EQ-5D Dimensions

Physically active male elderly subjects, when adjusted for gender, showed lower percentage of problems in all the dimensions. The dimension self-care (3.8% in male and 5.5% in female) is the one with the lowest percentage of problems, whereas the dimension pain/discomfort (48.4% in male and 72.1% in female) is the one with the highest percentage of problems in sedentary and physically active groups (Tables 2 and 3).

## 4. Discussion

People who engage in some kind of exercise have a significantly better perception of their health than the sedentary population in Catalonia. These data are coherent with most international [7, 8, 17–19] and national studies [9, 20]. Moreover, VAS absolute values in the case of physically active elderly are higher than 70, which implies good health [21]. This is not the case for measures obtained in the sedentary elderly. These data also show that there is an inverse relationship between age and HRQoL, which is coherent with the involution curve of physical skills and health in most epidemiological studies (ESCA'06).

Although the literature relates sports practice to health in a generalized way, positive relationships between level of physical practice and improvement of HRQoL are not always detected in all their dimensions. Rejeski et al. [8] found that in those areas where an elderly person has the same or higher functional level than that of a normal young adult, the impact of exercise is less important and measurement tools find it more difficult to detect changes. This is a fact that is more evident indeed when the variable is binary, as in the present study.

The dimensions of mobility, self-care, and usual activities showed a similar percentage of problems in the first age group (60–64 years), in both physically active and sedentary subjects. The difference becomes progressively bigger with age. The first age group under analysis, from 60 to 64 years, showed that physically active subjects present a higher percentage of problems than the sedentary groups in the dimensions of pain/discomfort and anxiety/depression, but this relationship is reversed with age (Tables 2 and 3). These data are coherent with studies that highlight the role of physical exercise as a protector from diseases and an enhancer of longevity [9].

The introduction of the SHC perspective in our results allows us to complement the previous findings from an economic perspective. Physically active elderly showed a higher SHC than sedentary subjects, which demonstrates their greater health potential.

The results showed that women's SHC is significantly higher than that of the physically active males, whereas in the case of sedentary subjects this relationship is reversed. We could interpret these results in the sense that physical activity improves women's health proportionally more in comparison with men.

## 5. Conclusions

The elderly people that engage in regular physical activity show a better HRQoL than the sedentary subjects, after adjusting for age and gender. Differences are more obvious in the older age groups. This better HRQoL can be seen both in a global assessment and in the different dimensions. The dimensions of mobility, usual activities, and self-care show an important evolution in the number of problems from 75 years old onward in the two groups that were analyzed. The dimensions of pain/discomfort and anxiety/depression show a higher percentage of problems for physically active elderly in the younger age groups, but this relationship is reversed as age increases. The measures of medication used corroborate results from a subjective perspective, representing better health for those people that engage in physical activity.

Positive assessments of physically active subjects, both from the perspective of VAS and DS, are directly correlated to a better SHC. As health improves with exercise, they have a higher LE; the SHC of physically active females improves in comparison to that of physically active males, who still have a lower LE than women.

We cannot deduce causal relationships among the factors analyzed. From this perspective, further longitudinal studies are needed to answer these questions.

Although results tend to relate an improvement in the health of subjects engaging in physical activity in comparison with sedentary subjects, it is noteworthy that in this study the subjects' health condition prior to measuring their HRQoL was not taken into account. In this respect, we think that further experimental research should consider this criterion or factor prior to intervention in order to confirm the present descriptive results.

Finally, we should highlight that the use of a generalist measurement tool such as the EuroQol, which was validated in Catalonia, has allowed us to compare a sample of subjects in this population. However, the use of an instrument that is not specific for a certain age group does not help to discriminate whether the main cause for worse health is fitness. Studies that work with homogenous age groups should help to refine our results.

## Figures and Tables

**Table 1:** Group statistics corresponding to the analysis of VAS, medication used and SHC by gender and age.

	Physically Active			Sedentary			*P*
	*N*	Mean	Standard Deviation	*N*	Mean	Standard Deviation	
*VAS *							
Male							
Global values	334	75.36	15.72	759	58.35	21.36	0.000
60 to 64 years old	52	76.81	15.46	155	62.50	23.03	0.000
65 to 74	184	74.95	15.36	270	59.47	22.35	0.000
75 to 84	96	74.91	16.46	256	56.43	18.55	0.000
Older than 85	2	97.50	3.54	78	52.56	21.44	0.000
Female							
Global values	1658	70.71	17.49	1115	50.59	21.25	0.000
60 to 64 years old	289	73.30	17.12	168	56.17	23.50	0.000
65 to 74	984	70.52	17.30	378	51.71	21.54	0.000
75 to 84	366	69.65	17.83	403	47.75	20.65	0.000
Older than 85	19	62.11	21.43	166	49.25	18.30	0.020
*Medication used *							
Male							
Global values	194	1.89	1.43	758	4.34	2.02	0.000
60 to 64 years old	33	1.21	1.11	155	4.12	2.24	0.000
65 to 74	102	1.96	1.52	270	4.16	1.96	0.000
75 to 84	57	2.19	1.33	255	4.68	1.95	0.000
Older than 85	2	1.00	1.41	78	4.36	1.87	0.171*
Female							
Global values	646	2.87	2.12	1114	4.21	2.24	0.000
60 to 64 years old	93	2.68	2.26	168	3.76	2.36	0.000
65 to 74	356	2.92	2.12	378	4.15	2.24	0.000
75 to 84	184	2.78	2.02	402	4.52	2.26	0.000
Older than 85	13	4.08	2.40	166	4.07	2.01	0.988*
*SHC *							
Male							
Global values	297	465988.31	150819.99	759	363689.33	230936.16	0.000
60 to 64 years old	51	671536.22	108351.82	155	600250.47	232833.02	0.003
65 to 74	163	484435.41	91804.46	270	429607.06	176906.70	0.000
75 to 84	81	306685.61	65474.50	256	229031.94	120407.96	0.000
Older than 85	2	172838.29	7480.72	78	107375.55	67194.41	0.000
Female							
Global values	1397	522550.31	212550.18	1115	346615.91	290873.26	0.000
60 to 64 years old	270	740420.35	194612.96	168	619035.23	319454.93	0.000
65 to 74	822	526876.34	167561.92	378	470962.52	280865.00	0.000
75 to 84	289	328150.82	111149.41	403	218492.30	167701.16	0.000
Older than 85	16	135084.22	87643.37	166	98810.80	79506.06	0.129*

*Mean differences were significant for *P* < 0.005. Assessment of every QALY = €34,858.70. Discount rate of 0%.

**Table 2:** Percentage of men with problems in EQ-5D dimensions according to age and physical activity level.

Mobility	60 to 64	65 to 74	75 to 84	Over 85	Total
Physically Active	12.7	18.7	17.2		17.2
Sedentary	27.0	32.0	58.3	77	45.1

Self-Care	60 to 64	65 to 74	75 to 84	Over 85	Total

Physically Active	3.6	4.8	2.1		3.8
Sedentary	10.2	9.2	23.2	34.2	15.9

Usual Activities	60 to 64	65 to 74	75 to 84	Over 85	Total

Physically Active	7.4	9.0	3.1		7.0
Sedentary	20.0	20.4	39.5	56.1	29.3

Pain/Discomfort	60 to 64	65 to 74	75 to 84	Over 85	Total

Physically Active	47.2	47.9	50.0	50.0	48.4
Sedentary	38.7	52.4	61.8	59.0	52.7

Anxiety/Depression	60 to 64	65 to 74	75 to 84	Over 85	Total

Physically Active	18.2	18.2	19.1		18.3
Sedentary	26.5	24.8	26.6	21.8	29.4

**Table 3:** Percentage of women with problems in EQ-5D dimensions according to age and physical activity level.

Mobility	60 to 64	65 to 74	75 to 84	Over 85	Total
Physically Active	19.2	28.6	28.6	47.6	27.1
Sedentary	43.7	59.6	72.7	82.1	64.4

Self-Care	60 to 64	65 to 74	75 to 84	Over 85	Total

Physically Active	4.5	4.8	7.1	25.0	5.5
Sedentary	16.3	22.8	35.9	45.5	29.2

Usual Activities	60 to 64	65 to 74	75 to 84	Over 85	Total

Physically Active	14.6	20.8	24.7	30.4	20.7
Sedentary	32.1	45.2	61.4	70.0	51.8

Pain/Discomfort	60 to 64	65 to 74	75 to 84	Over 85	Total

Physically Active	64.8	73.6	74.0	69.6	72.1
Sedentary	69.0	76.7	80.8	86.2	70.8

Anxiety/Depression	60 to 64	65 to 74	75 to 84	Over 85	Total

Physically Active	38.7	37.4	34.1	40.0	37.0
Sedentary	52.4	46.5	48.1	46.4	48.0
